# ABO blood group system and placental malaria in an area of unstable malaria transmission in eastern Sudan

**DOI:** 10.1186/1475-2875-6-110

**Published:** 2007-08-10

**Authors:** Ishag Adam, Saud Babiker, Ahmed A Mohmmed, Magdi M Salih, Martin H Prins, Zaki M Zaki

**Affiliations:** 1 Department of Obstetrics & Gynecology, Faculty of Medicine University of Khartoum, Sudan; 2 Faculty Of Medical Technical Sciences, Alzaiem Alazharai University, Sudan; 3 Faculty of Medicine, Ribat University, Sudan; 4 Faculty of Medical Laboratory Sciences, University of Khartoum, Sudan; 5 Department of Epidemiology, Care and Public Health Research Institute, University of Maastricht, The Netherlands

## Abstract

**Background:**

Understanding the pathogenesis of malaria in pregnancy and its consequences for both the mother and the baby is fundamental for improving malaria control in pregnant women.

**Aim:**

The study aimed to investigate the role of ABO blood groups on pregnancy outcomes in an area of unstable malaria transmission in eastern Sudan.

**Methods:**

A total of 293 women delivering in New Half teaching hospital, eastern Sudan during the period October 2006–March 2007 have been analyzed. ABO blood groups were determined and placental histopathology examinations for malaria were performed. Birth and placental weight were recorded and maternal haemoglobin was measured.

**Results:**

114 (39.7%), 61 (22.1%) and 118 (38.2%) women were primiparae, secundiparae and multiparae, respectively. The ABO blood group distribution was 82(A), 59 (B), 24 (AB) and 128 (O). Placental histopathology showed acute placental malaria infections in 6 (2%), chronic infections in 6 (2%), 82 (28.0%) of the placentae showed past infection and 199 (68.0%) showed no infection. There was no association between the age (OR = 1.02, 95% CI = 0.45–2.2; *P *= 0.9), parity (OR = 0.6, 95% CI = 0.3–1.2; *P *= 0.1) and placental malaria infections. In all parity blood group O was associated with a higher risk of past (OR = 1.9, 95% CI = 1.1–3.2; *P *= 0.01) placental malaria infection. This was also true when primiparae were considered separately (OR = 2.6, 95% CI = 1.05–6.5, *P *= 0.03).

Among women with all placental infections/past placental infection, the mean haemoglobin was higher in women with the blood group O, but the mean birth weight, foeto-placental weight ratio was not different between these groups and the non-O group.

**Conclusion:**

These results indicate that women of eastern Sudan are at risk for placental malaria infection irrespective to their age or parity. Those women with blood group O were at higher risk of past placental malaria infection.

## Background

It has been estimated that 90% of the global malaria burden occurs in sub-Saharan Africa, where 40% women are exposed to malaria infections during pregnancy [1]. Malaria during pregnancy poses a substantial risk to the mother, her foetus and the neonate [2]: it is a major health problem in Sudan, where it has been reported to be associated with maternal anaemia, low birth weight infants and a major cause of maternal mortality [3-6].

Host genetic factors modulate the risk and severity of infection via specific mediators, which can be different in various epidemiological settings. Cell adhesion plays a fundamental role in placental malaria pathophysiology [7]. Cell surface glycans, such as the ABO and related antigens could modulate some of those specific cell interactions [8]. A century since the discovery of the ABO blood groups, numerous associations between ABO groups and disease have been reported. Interestingly, some strains of *Plasmodium falciparum *preferentially trigger rosette formation depending on the red blood cell blood group [9]. However, the rosette formation is not a feature of *P. falciparum *infections during pregnancy [10].

Recently, ABO blood groups have been reported to be one of the host risk factors for placental malaria infections and they are associated with other materno-foetal outcomes [11]. The current study was conducted in eastern Sudan, an area that is characterized by unstable malaria transmission [12] and where malaria is a substantial burden affecting pregnant women irrespective to their age or parity [3]. The study aimed to investigate the epidemio-pathogical characteristics of placental malaria infections with special attention to blood groups and their association with materno-foetal outcomes and to add to ongoing data on the pathogenesis of malaria during pregnancy in the area [13].

## Methods

### Patients

The study was conducted between October 2006 and March 2007, at the labour ward of New Halfa Teaching Hospital, eastern Sudan. After informed consent, women with a singleton baby were approached to participate in the study. Those with antepartum haemorrhage, hypertensive disorder of pregnancy (diastolic blood pressure > 90 mm Hg) and diabetes mellitus were excluded. A structured questionnaire was administered to collect information about socio-demographic characteristics, mode of delivery and birth outcome. Obstetrical examinations were performed and the findings recorded (blood pressure, pallor, fundal level and foetal heart sound). The babies were weighted immediately following birth on a Salter scale to the nearest 10 g; scales were checked for accuracy on a weekly basis.

### Haematology

Maternal, placental and cord blood films were prepared, the slides were Giemsa-stained and the number of asexual *P. falciparum *parasites per 200 white blood cells was counted and double-checked blindly by an expert microscopist. Maternal haemoglobin concentrations were estimated by HemoCue haemoglobinometer (HemoCue AB, Angelhom, Sweden). Maternal blood groups were investigated by the agglutination method.

### Histopathology

Full thickness placental blocks of around 2–3 cm were taken from the placenta, kept in neutral buffer formalin for histopathology examinations. Placental malaria infections were characterized based on the classification of Bulmer *et al *[14]: uninfected (no parasites or pigment), acute (parasites in intervillous spaces), chronic (parasites in maternal erythrocytes and pigment in fibrin or cells within fibrin and/or chorionic villous syncytiotrophoblast or stroma), past (no parasites and pigment confined to fibrin or cells within fibrin).

### Statistics

Data were entered in computer using SPSS for windows and double-checked before analysis. Means and percentages were compared either by Students' *t*-test, ANOVA (if data were normally distributed) or by the Mann-Whitney test (if the data were not normally distributed). X^2 ^and Fisher's exact tests as appropriate. Multiple linear regressions were used to analyse placental malaria infections as a dependent variable and age ≤ 19 years, parity (primiparae, secundiparae and multiparae) and maternal blood group (O versus non O) as possible influencing factors. The others factors included in the analyses were: maternal blood group (O versus non O), parity, placental infection, maternal haemoglobin, birth weight and foeto-placental ration. *P *≤ 0.05 was regarded as significant.

### Ethics

The study received ethical clearance from the Research Board at the Faculty of Medicine, University of Khartoum.

## Results

During the study period, there were 578 deliveries. Of these, 24 were twins and four were triplets, 550 singleton deliveries. The data of placental histopathology, maternal haemoglobin, placental and birth weight were complete in 293 women; these data were included in the final analyses. Out of these 293 women, there were 114 (39.7%), 61 (22.1%) and 118 (38.2%) primiparae, secundiparae and multiparae, respectively. The mean (SD) age was 25.6 (6.4) years and 57(19.4%) of the women were teenagers (age ≤ 19 years). Of the 293 women, 82 (28.0%), 59 (20.1%), 24 (8.2%) and 128 (43.7%) had blood group A, B, AB and O, respectively. The mean (SD) haemoglobin was 11. 4 (2.1) gm/dl. The mean (SD) birthweight was 3,063.4 (545.4) gm, while the mean foeto/placental ratio was 5.6 (1.2). Maternal and placental blood films were positive in four cases and the blood films for malaria were positive in one maternal, placental and cord setting.

Six (2%), 6 (2%) and 82 (28.0%) of the placentae showed acute, chronic, past infection on histopathology examination respectively, while 199 (68.0%) of them showed no infection. There were no associations between the age (OR = 1.02, 95 CI = 0.45–2.2; *P *= 0.9), parity (OR = 0.6, 95 CI = 0.3–1.2; *P *= 0.1) and placental infections (all or in subgroups).

Women who have blood group O were at higher risk for past placental infections (OR = 1.9, 95 CI = 1.1–3.2; *P *= 0.01). This was also true when primiparae were considered separately (OR = 2.6, 95% CI = 1.05–6.5, *P *= 0.03 (Table 1).

**Table 1:** ABO phenotype by placental malaria category and parity group

Parity	Placental malaria		Blood group Phenotype			
	Type	number	A	B	AB	O

Total	Acute	6	5 (6.1)	1(1.7)	0 (0)	0 (0)
	Chronic	6	2(2.4)	1(1.7)	1(4.2)	2(1.6)
	Past	82	19(23.2)	13(22.0)	5(20.8)	45(35.2)*
	None	199	56 (68.3)	44(74.6)	18(75)	81(63.3)
	Total	293	82	59	24	128
Primiparae	Acute	2	2(6.5)	0(0)	0(0)	0(0)
	Chronic	1	0(0)	0(0)	1(12.5)	0(0)
	Past	26	5(16.1)	3(12.5)	2 (25.0)	16(31.4)*
	None	85	24(77.4)	21(87.5)	5(62.5)	35(68.6)
	Total	114	31	24	8	51
Secundiparae	Acute	2	1(4.2)	1(12.5)	0(0)	0(0)
	Chronic	0	0(0)	0(0)	0(0)	0(0)
	Past	18	8(33.3)	0(0)	0(0)	10(38.5)
	None	41	15(62.5)	7(87.5)	3(100)	16(61.5)
	Total	61	24	8	3	26
Multiparae	Acute	2	2(7.4)	0	0	0
	Chronic	5	2(7.4)	1(3.7)	0	2(3.9)
	Past	38	6(22.2)	10(37)	3(23.1)	19(37.3)
	None	73	17(63.0)	16(59.3)	10(76.9)	30(58.8)
	Total	118	27	27	13	51

* Significant difference

Past placental malaria infections were observed in 45 (35.2%) of 128 women with blood group O versus 37 (22.4%) of 165 with non-O group, *P *= 0.0 1. This was also true when primiparae were considered separately, 16 (31.4%) of 51 primiparae women with group O versus 10 (15.9%) of 63 with non-group O, *P *= 0.05, (Table 2).

**Table 2:** Mean (SD) maternal and birth outcomes by parity and maternal blood group phenotype

Parity	Maternal and foetal parameters					
	Placental malaria type	Blood group	number	Maternal Hb	Birth weight	Foeto-placental ratio

All parity	Infection	Group O	47	11.8(2.1)*	3060.1(370.0)	5.4(1.0)
		Non-O	47	10.9(1.9	310.9(575.4)	5.6(1.0)
	No infection	Group O	81	11.5(2.1)	3098.3(561.6)	5.7(1.1)
		Non-O	118	11.6(2.2)	3037.2(554.2)	5.6(1.4)

Primiparae	Infection	Group O	16	12.0(2.0)*	2946.4(367.2)	5.6(0.8)
		Non-O	13	10.4(2.3)	2898.3(409.7)	5.7(0.9)
	No infection	Group O	35	11.1(2.1)	3065.4(462.4)	5.8(1.0)
		Non-O	50	11.7(2.3)	3019.8(593.9)	5.8(1.8)

Secundiparae	Infection	Group O	10	11.1(2.5)	3150.1(348.4)	5.7(0.3)
		Non-O	10	11.0(1.2)	3298.6(655.0)	5.5(1.3)
	No infection	Group O	16	11.5(1.9)	3251.2(570.5)	6.0(1.2)
		Non-O	25	12.1(2.1)	2947.4(529.9)	5.4(1.0)

Multiparae	Infection	Group O	21	12.0(2.0)	3098.5(379.2)	5.1(0.98)
		Non-O	24	11.1(2.0)	3038.0(604.4)	5.5(1.3)
	No infection	Group O	30	11.9(2.1)	3055.6(656.9)	5.4(1.1)
		Non-O	43	11.2(2.0)	3109.524.8)	5.3(0.9)

* Significant difference

The mean birth weight and foeto-placental ratio were not significantly different between those with and without placental malaria infections or those women with blood group O and non-O group, (Table 2).

Maternal haemoglobin concentration was significantly higher in O type mothers with all placental/past placental malaria (11.8 g/dl, versus 10.9 g/dl, *P *= 0.02), this difference was statistically significant even when primiparae were considered separately (12.0 g/dl, versus 10.4 g/dl, *P *= 0.05), (Table 2).

## Discussion

As in previous findings on the peripheral microscopically- and submicroscopically-detected parasitaemia and the pathogenesis of malaria during pregnancy in the same hospital [3,13,15], the histopathological placental malaria infections were not associated with age or parity in the current study. The explanation for this may be the low immunity in the area, which is characterized by unstable malaria transmission [12].

In this study around 32% of the placentae had been observed to have some degree of infections, few of these infections were acute while the vast majority was past infections. Women (even primiparae) with blood group O were at higher risk for past placental malaria infections. While significantly higher haemoglobin was observed in women with past placental malaria and blood group O, the other parameters (birth weight and foeto-placental ration) were not different between the two groups. Surprisingly, in this study the birthweight was not associated with histopathological placental malaria infections and the explanation for this remains unclear. These women had very low prevalence of peripheral, placental and cord microscopically detected parasitaemia, hence the role of submicroscopic parasitaemia on low birthweight cannot be excluded. Previously, a large burden of submicroscopically-detected parasitaemia was observed to be associated with morbidity and mortality among pregnant and non-pregnant individuals in the same geographical area [15,16].

Recently, Loscertales and Brabin [11] have observed that; in primiparae, blood group O was associated with a higher risk of acute infection and a lower risk of past infection, in contrary to multiparae in whom O phenotype was associated with reduced prevalence of acute or past placental infection. However, findings from other studies suggested that individuals of blood group O are relatively resistant to severe disease caused by *P. falciparum *infection. It was established that parasitized erythrocytes form rosettes more readily with red blood cells (RBCs) of A, B, or AB groups than with blood group O and this parasite-triggered RBC rosette formation is associated with the severity of clinical disease and with the development of cerebral malaria [17,18]. Pregnant women are more attractive for the vector [19] – and a differential affinity of blood group O for the vector has also been reported before [20]. Furthermore, an antigen sharing between ABO phenotypes and *P. falciparum *leading to changes in immune response [21] or facilitating entry of the parasite into the red cell has been described recently [22].

In the current study, women with past placental infection had a higher mean haemoglobin when they were of O blood group. This correlates with the previous findings where non-pregnant patients with severe *P. falciparum *malaria and non-O blood group had significantly lower haemoglobin levels compared with patients with group O blood [23]. The higher Hb level associated with the O phenotype could relate to the development of improved malaria immunity in this particular group of women, following on from a preceding increased risk of malaria infection in this phenotype. Recently, Loscertales and Brabin [11] have also reported higher (although, not statistically significant) haemoglobin in women with blood group O.

## Conclusion

These results indicate that women of eastern Sudan are at risk for placental malaria infection irrespective to their age or parity. Those with blood group O were at higher risk of past-chronic placental malaria infection. Among women with past-chronic placental infection, the mean haemoglobin was higher in women with the O blood group, but the mean birthweight and foeto-placental weight ratio were not significantly different between these groups.

## Authors' contributions

IA and SB carried out the study and participated in the statistical analysis and procedures, ZMZ participated in the statistical analysis, MP coordinated and participated in the design of the study, statistical analysis and the drafting of the manuscript. AM and MS participated in the lab work. All the authors read and approved the final version.
